# What explains between-school differences in rates of sexual experience?

**DOI:** 10.1186/1471-2458-8-53

**Published:** 2008-02-08

**Authors:** Marion Henderson, Isabella Butcher, Daniel Wight, Lisa Williamson, Gillian Raab

**Affiliations:** 1 MRC Social and Public Health Sciences Unit, Glasgow, UK; 2 Public Health Sciences, University of Edinburgh, Edinburgh, UK; 3 School of Nursing, Midwifery & Social Care, Napier University, Edinburgh, UK

## Abstract

**Background:**

Schools have the potential to influence their pupils' behaviour through the school's social organisation and culture, as well as through the formal curriculum. This paper provides the first attempt to explain the differences between schools in rates of reported heterosexual sexual experience amongst 15 and 16 year olds. It first examined whether variations in rates of sexual experience remained after controlling for the known predictors of sexual activity. It then examined whether these residuals, or 'school effects', were attributable to processes within the school, or were more likely to reflect characteristics of the neighbourhood.

**Methods:**

Longitudinal survey data from 4,926 pupils in 24 Scottish schools were linked to qualitative and quantitative data on school processes including quality of relationships (staff-pupil, etc), classroom discipline, organisation of Personal and Social Education, school appearance and pupil morale. Multi-level modelling was used to test a range of models and the resulting 'school effects' were then interpreted using the process data.

**Results:**

Overall, 42% of girls and 33% of boys reported experience of sexual intercourse, with rates by school ranging from 23% to 61%. When individual socio-economic and socio-cultural factors were taken into account the school variation dropped sharply, though pupils' attitudes and aspirations had little effect. There was very little correlation between boys' and girls' rates of sexual experience by school, after controlling for known predictors of sexual activity. Girls were more influenced by individual socio-economic factors than boys. School-level socio-economic factors were predictive even after taking account of individual socio-cultural factors, suggesting that the wider socio-economic environment further influenced young people's sexual experience.

**Conclusion:**

Importantly, school processes did not explain the variation between schools in sexual experience. Rather, the variation may have been due to neighbourhood culture.

## Background

Among teenagers living in Scotland, between 31% and 33% of young women and between 26% and 28% of young men have had sexual intercourse by their sixteenth birthday [1,2]. These rates, alongside concern about HIV/AIDS and conceptions among girls under 16, have made young people's sexual health a priority public health issue [3-5]. Given that the vast majority of young people attend school until at least age 16, it is important to explore and understand the possible influence of schools on rates of young people's sexual experience. This paper is based on data from a randomised trial, which found that formal sex education did not influence behavioural outcomes [6]. We are, therefore, turning our focus to the potentially broader impact of schools through mechanisms other than the formal curriculum. This has been investigated through research into 'school effects'.

A 'school effect' means 'where pupil outcomes for a school vary, either positively or negatively, from that which might be expected given the known predictors of these outcomes' [7]. The study of 'school effects' on academic outcomes has a long history, but their impact on health-related behaviours has received less attention and we are not aware of any formal 'school effects' research relating to sexual experience (although there are some papers that discuss related concepts that will be cited later). This paper will help to address the gap with regard to 'school effects' on sexual experience, in order to explore whether schools influence pupils' health behaviours not just through the formal curriculum, but also through the social organisation and culture of the school.

### What is known about 'school effects'

Until twenty-five years ago, research such as the Coleman Report [8] was widely interpreted as concluding that schools have little or no effect on student achievement, when family background variables have been taken into account. Subsequent work has challenged these conclusions [9,10]. Factors were identified within schools that determined high levels of school effectiveness across a wide range of mainly academic outcomes [10]. More recently, detailed observation of schools generated a wide range of data on schools that revealed a number of factors that were associated with more effective schools: a high proportion of pupils in authority positions, a low level of institutional control, positive academic expectations, a low level of coercive management, a high level of student involvement, small overall size of school, low teacher/pupil ratios in classes, and tolerant attitudes to the enforcing of certain rules regarding dress, manners and morals [11,12].

Though the studies described above suggest a number of different mechanisms by which schools might affect outcomes, there is some overlap. They all suggest that pupils should be treated as if they are responsible, that there should be academic goals, and that management should be democratic. Furthermore, in an overview of the 'school effectiveness' literature [12] it was stated that the findings are applicable over a wide range of student outcomes, so it is of interest to establish if this extends to sexual behaviour.

### Theory to link school characteristics to health behaviours

Currently, the Health Promoting School (HPS) concept [13] is accepted as a theory that guides school health promotion practice internationally [14,15]. It requires that schools move beyond their formal health education curricula to examine how their policies and practices throughout the school, in particular quality of social relationships, affect the health and well-being of pupils. The guidelines developed to achieve this are based on the philosophy that:

'The Health Promoting School aims at achieving healthy lifestyles for the total school population by developing supportive environments conducive to the promotion of health. It offers opportunities for, and requires commitments to, the provision of a safe and health-enhancing environment.' [13]

The definition raises awareness of both social and physical environments. The HPS concept is very compatible both with the concept of a 'positive climate' [16] and work on school ethos [17]. The concept of school ethos was encapsulated by twelve components (including quality of relationships, communication and physical environment) in order that schools can measure their progress towards achieving a positive ethos [17].

The 'school effect' literature described above is also compatible with the HPS concept in that both emphasise the importance of quality of social relationships and communication in order to have whole school practices that are cohesive. However, while the 'school effects' research (relating primarily to academic outcomes) has identified links between processes and outcomes, to date such links are at an early stage for the HPS concept. Until recently the focus was on provision and process, but recently evidence for specific outcomes has started to be published [15,18].

Schools in Scotland and England are being encouraged to become HPS [19,20]. There is growing evidence that the HPS approach works for mental health, violence and smoking, with mixed evidence for drug use, diet and physical activity [18,21-24]. However, there is limited evidence whether this approach may be effective for sexual health. This paper aims to make a contribution to this area.

### Schools and sexual behaviour

A recent literature review cites correlational studies that highlight that greater levels of involvement in school (namely, investment in school (by good attendance and/or not dropping out), involvement in school, attachment to school and school performance) are associated with older initiation of first sexual intercourse, lower frequency of sex and less pregnancy [25]. Similarly, a U.S.A. based longitudinal study in adolescent health found an association between delay in sexual debut and three factors, namely, higher levels of school connectiveness, attending a parochial school and attending a school with high overall average daily attendance (adjustment was made in the analysis for poverty) [26]. Finally, the Safer Choices programme was a schoolwide intervention which aimed to influence school climate and was successful at reducing unsafe sex and pregnancy [27]. These studies support the idea that school characteristics have the potential to influence sexual behaviour.

This paper will use data collected as part of a randomised trial of school sex education[6]. The variables predicting sexual experience for pupils in this trial have previously been demonstrated, for age 14 [28] and age 16 [29]. The variables were family composition, levels of parental monitoring, amount of personal spending money, mother's qualifications, mother's social class, mother's age, father's qualifications, father's social class, housing tenure, ethnic group, and strength of religious belief. These variables will be adjusted for before 'school effects' are deemed to exist (i.e. these are the 'known predictors' of sexual experience for this sample). The paper will then establish a) whether there are demonstrable 'school effects' on levels of pupils' sexual experience, and b) if so, whether these 'school effects' are attributable to identifiable school characteristics such as quality of relationships, attitudes to school, physical environment and the academic focus of the school. Answering these questions will extend current knowledge in this field.

## Methods

### Background

Ethical approval for a randomised trial of a specially-designed school sex education programme (SHARE) was gained from Glasgow University's Ethical Committee for Non-Clinical Research Involving Human Subjects. SHARE is a teacher-delivered sex education programme that differs from conventional sex education in devoting a third of the 20 sessions to developing sexual negotiation and condom skills [30]. All non-denominational state schools within 15 miles of the main cities in Tayside and Lothian regions of South-East Scotland (excluding schools involved in the pilot studies) were invited to participate (N = 47). Twenty-five schools agreed to take part and were allocated to either implement the SHARE programme or continue with their existing sex education, according to a balanced randomisation. The main reasons the 22 remaining schools gave for not participating were the practical difficulties envisaged in implementing SHARE (e.g. timetabling or mobility of teachers), although a few referred to the explicit nature of the programme and research.

Pupils' parents and the pupils themselves were informed about the longitudinal study (trial) and given the opportunity to withdraw.

### Pupil sample

During 1996 and 1997 two successive cohorts of 13 – 14 year olds participated in a baseline survey (mean age 14 years and two months). These 7,616 pupils who participated (of the 8,430 eligible) were representative of 14 year olds throughout Scotland, in terms of parents' social class and the proportion of one-parent households, using 1991 Census data[28] This paper also uses data collected in 1998 and 1999 at the first follow-up of the SHARE trial, when the cohorts were aged 15 or 16 (mean age 16 years and one month). By this age 23% had left school. Follow-up data were collected from 5,854 young people giving an overall participation rate of 70%. There was a very different participation rate for those still at school (81%) and early school leavers (39%). A small proportion (2%) refused to participate (Wight et al., 2002). Early school leavers are those that leave school as soon as they are legally old enough to do so (16 years of age). Leaving that early will very rarely have enabled the young person to have sat the level of examinations that would allow access to Higher Education such as University/College.

One school chose not to participate at baseline and is therefore excluded from our analysis (380 pupils). Further exclusions were applied to cases with high levels of missing data (362 pupils), with incomplete data on important covariates (118 pupils), or where we were not certain that sexual intercourse had taken place (68 pupils).

In this paper we have used data from 4,926 pupils for whom we have both baseline and follow-up data (65% of those that originally participated at baseline). These pupils are included in the all statistical models described.

### Pupil level measures

The pupil questionnaires at baseline and age 16 follow-up broadly asked the same questions, although the age 16 questionnaire was slightly longer. The questionnaires (and additional information about SHARE) are available on a public domain Internet site [31].

The questionnaire covered the following topic areas: questions about pupils' sexual experience; socio-cultural variables (e.g. family composition and parental monitoring); attitudinal variables (e.g. attitude to school and self-esteem), aspirational variables (e.g. what the young people think they will be doing in 2 years time); and an indicator of proportion of friends at other schools and proportion of friends who have left school.

For sexual experience, pupils were told that, 'in questions that follow 'sexual intercourse' means: a boy/man putting his penis into a girl/woman's vagina, or 'going the whole way'. The pupils were then asked 'Have you experienced any of the following with a girl/woman [or boy/man]? Then the pupils could tick yes or no to sexual intercourse. As part of the data cleaning process we examined the logical consistency of the answer with reference to ten further questions about first sexual intercourse. All pupils included in this analysis had logically consistent answers (68 were excluded on this basis, see also above).

### Procedures with pupils

The pupil questionnaires took approximately 50 minutes to complete. For the young people still at school (all participants at baseline and those still at school at follow-up), questionnaires were administered by trained researchers in classrooms in exam conditions (i.e. pupils had privacy and quiet to complete the questionnaires), at the start of the fifth year. Teachers left the room before pupils started answering questionnaires, and these were sealed in envelopes once completed. Questionnaires had identity numbers but not respondents' names. Absentees were left questionnaires to complete in school and return by post, while persistent non-attendees and those who had left school at follow-up were contacted at home by post.

### Process measures

Process data reflect the characteristics of the school, including information on relationships between different dyads within the school (e.g. teacher-pupil, pupil-pupil and teacher-teacher), but also appearance, discipline and layout of the school.

Data on processes within schools were collected through pupil questionnaires, interviews and group discussions, teacher questionnaires and interviews, ethnographic notes, classroom observation and fieldworkers' observations [32]. Four kinds of data are used in this analysis. First, there are individual-level data from pupil questionnaires about the degree to which the respondent likes school (2 items), and teachers trust and respect pupils (2 items). Second, there are school-level data from questionnaires with sex education teachers (N = 151) about senior management to staff relationships (1 item), staff to staff relationships (2 items) and staff to pupil relationships (2 items). The Cronbach's Alpha for all these items derived independently from pupils and teachers was over 0.7. Focusing solely on staff-pupil relationships, as reported independently by teachers and pupils, yielded a Cronbach's Alpha over 0.9. These Cronbach's Alpha scores indicate that, despite being reported independently by teachers and pupils and through different questionnaire items, there is good internal consistency across the items, demonstrating the validity of this information. These items were included in the factor analysis described below.

The third kind of process information is qualitative, arising from 58 in-depth teacher interviews (conducted with at minimum, the teacher responsible for sex education in each school), observations of lessons with 41 teachers (in 15 schools) and numerous ethnographic notes (from all schools). The interviews covered, amongst other topics, relationships between sex education colleagues, support from senior management, staff-pupil relationships and perceived ethos of the school. Amongst other things systematically noted in the lesson observations were: teacher-pupil relationships, pupil-pupil relationships and pupil behaviour. Two qualitative researchers (including DW) coded these data and then reviewed all the relevant information pertaining to nine aspects of the school, giving a General Score (GS) to each on a scale of 1 (poor), 2 (OK) to 3 (good). The aspects were: pupil morale, relationships between staff and pupils, staff and staff, and staff and senior management, academic focus, organisation of PSE, discipline, school-home relationships and physical environment (Table 1). A second researcher validated this scoring and any discrepancies were discussed with a third researcher (MH) who had also frequently visited the schools until a consensus score was agreed. Inter-rater reliability scores were not calculated, however discrepancies in scoring were usually of only one point, and only in one school were there discrepancies of two points. In establishing consensus scores the researchers drew on their wider knowledge of the teacher, pupils or school in order to better contextualise the recorded observations. It should be noted that the quantity and type of information held about each school varied considerably, and for all but one of the variables there were missing data for some schools.

**Table 1:** Summary of process data for schools

School	Rank for boys	Rank for girls	RP: staff-pupil rel.	RP: staff-staff rel.	RP: pup.-pup. Relat.	RP: Appearance	RP: Discipline	RP: layout of school	GS: Pupil morale^1^	GS: Pupil-staff relat.	GS: Academic focus	GS Organised PSE	GS: Discipline	GS: Staff-staff relat.^2^	GS: Staff-sen. man. rel.	GS: School-home rel.	GS: Physical envir.
9	1	24	4.4	4	4.4	4	4.5	2			(U) 2/3	Reg.	1/2	3	3		1/2
15	2	3	3.7	3.3	3.9	3.9	4	3.2	3		NU 1/2	Reg.	3			3	2
6	3	14	3.9		4.2		4.4		1		U 3	Reg.	1			1	
17	4	6	3.8	4.3	3.8	2.6	4.1	3.7			NU 3	Reg.	2	1	1	3	3
24	5	15	4.1	2.2	3.7	3.1	3.4	3	2	1	U 3	Reg./French		1	1		1
10	6	19	3.9	4.2	4.3	5	4.2	5	3		U* 1	Reg.		3			3
3	7	7	3.1	2	3.8	3.6	4.4		1		(U)	1st French 2nd Maths	1		1		1
14	8	8	3.7	4	4	4.6	4.5	4.2	3		U 1	Reg.	3			3	3
8	9	16	3.8	2	4	2.7	4.1	3			3	Reg./French				1	1/2
2	10	5	4.6	4.4	5	4	4.5	4			NU 3	Reg./French	1	1			
1	11	17	4.5	4	4.2	2.6	4.2	2.3	3	3	U 1	Reg.		3	3	3	3
22	12	11	4.5	4.5	4.4	3.4	4.4	3.8	2		U	English sets	3	2	1		
12	13	21	3.8	4	4	3	3.9	2.3			U	Reg/French	1/2	2			
23	14	2	3.2	4.4	3.8	4.1	4	3.5			U* 1	Alphabetical	3			3	
21	15	23	3.5	3.3	4.3	3.7	4.1	2.7		1/2	U* 1	Reg.	3	2	2		1
18	16	12	4.3	2.5	3.9	3.2	4.1	2.7			U* 1	Reg./RE/PE	2			1	2
16	17	19	3.9	3.9	3.9	3	3.5	3			(NU) 2	Reg./French	1	3	1		1/2
19	18	20	3.5	4.7	4	3.5	3.7	3.8		3	U 3	Reg./French	1	3	3	1	1/2
11	19	18	3.7	5	4.2	4.5	4.1	4.9			U 1/2	Maths sets		1/2		3	
5	20	1	3.1	2.6	3.5	2.5	3.3	2.8	1	1/2	U 1/2	Reg./French	1	1	1	1	1
7	21	13	3.4	3.7	4.1	3.9	4.2	3			U 1	English		3	2	3	
25	22	4	4.3	2.9	3.2	2.7	3.9	2.9		3	U 3	Reg./French	1/2	3		1	1
13	23	22	4	4.4	4.1	3.7	4	3			U*	1st Eng. sets 2nd not set			1	1	
20	24	2	3.8	3.4	4	4.1	4	3.3	1		U 3	Reg.	1	1	1		2
	MEAN		3.8	3.8	4	3.6	4	3.3									

With Researcher Perceptions (RP) measures mean scores from 1996 and '97 data: 5 best, 1 worst.

With 'Academic focus': 1 focus on academic achievement, 2 in-between, 3 focus on caring and inclusiveness. NU no uniform, (U) probably a uniform policy but hardly anyone wears it, U uniform, U* clear uniform that nearly everyone wears.

General scores (GS): 1 poor, 2 OK/average, 3 good.

^1 ^Pupil-pupil relations, bullying, extent to which they enjoy school, promotion of self-esteem by involving pupils in school life

^2 ^Relationships between staff and general staff morale

Fourth, and finally, process data were collected during each class visit to collect survey data. Researchers scored all classes visited according to six variables: staff to pupil relationships, staff to staff relationships, pupil to pupil relationships, discipline, appearance of the school, and layout of the school. The ratings were on a scale of 1 (low) to 5 (high). These "researchers' perceptions" involved scores from around 20 different researchers which were then averaged for each school (Table 1). Data were collected from over 400 school classes which, in the majority of cases, were administered by one researcher, however, for 10% of classes there were two researchers present who each rated the classes. During training the criteria for the ratings were discussed and then new researchers shadowed a more experienced researcher during their first two school visits. During shadowing both researchers were asked to make their rating independently and then these were discussed for training purposes. All the Cohen's Kappa's for inter-rater reliability, for instances where two researchers rated the same school classes, were above 0.7. This indicates substantial inter-rater reliability above chance and good enough to proceed with analysis. Given that the paper is discussing school effects and not class effects, the ratings for each of the classes within a school were averaged. As we have confidence in the inter-rater reliability for each class, averaging to school level should provide an estimate of where each school lay compared to the others.

The school-level process data of the first and second kind are used within the modelling procedures to explain the residuals. The third and fourth kind of data are used externally to the modelling to try and further explain the residuals.

### Statistical analysis including preliminary analysis

The main outcome examined in this investigation was a binary indicator of reported experience of sexual intercourse at time of follow-up. This was used in preference to use of the baseline reports of sexual intercourse for several reasons. Baseline data were not available for all pupils and the evidence suggested that some reports were unreliable [28]. Further, the rates of sexual experience at baseline were low (18% for boys and 15% for girls). To explore 'school effects', a series of models were fitted to the data. These allowed us to examine the results before and after adjustment for pupil and school characteristics. Comparing 'school effects' between models revealed which factors contributed to differences between schools.

School level data were also incorporated in the modelling to ascertain whether they helped to explain the school effect. Data on thirteen variables had been collected at the outset of the trial to facilitate balanced randomization of schools [33]. Principal components factor analysis was carried out to reduce the dimensionality of these data. This analysis identified 4 factors with eigenvalues greater than 1, accounting for around 80% of the variability. One variable, a composite measure of school ethos, was seen to be contributing roughly equally to 2 of the factors, so was deemed to be a contaminant, and thus removed from the analysis. In the resulting rotated factor solution it was found that the 7 deprivation-related variables -unemployment in school catchment area, deprivation score of local area, pupils' post school destination, proportion receiving free school meals, staying-on rates (S4 to S5 and S5 to S6), and attendance rates – were grouped together in the first factor. The second factor was dominated by the variables denoting access to clinics and the number of placement requests for a school (this picks out the urban/rural areas). Pupil-rated teacher-pupil and teacher-teacher relationships comprised the third factor and a proxy for school size in the fourth. Only factor one, which had high loadings for 7 of the thirteen variables, proved to be significant in explaining residuals in the levels of sexual experience at the school level. All 7 variables contributing to the significant factor were related to the deprivation of the school, with higher values being associated with more affluent schools. Thus only this factor was included as a school-level component at the stage of Model 5 (see below).

A two-level logistic regression model with pupils at level one and schools at level two was used. Computations were carried out using the GLMMIX macro in SAS Version 8.1 [34]. This was done separately for boys and girls.

The modelling was carried out in stages, adding groups of individual level variables to a basic model as follows:

**Model 1** : Basic model: pupils' age in months at follow-up and cohort

**Model 2** : Model 1 plus individual socio-cultural variables (who the young person lived with, levels of parental monitoring, amount of personal spending money, mother's qualifications, mother's social class, mother's age, father's qualifications, father's social class, housing tenure, ethnic group, and strength of religious belief). The importance of these factors in predicting sexual experience has previously been demonstrated, for age 14 and age 16 [28,29].

**Model 3** : Model 2 plus attitudinal variables (self-esteem, attitude to school, pupil-assessed teacher-pupil relationships, and proportion of peers perceived to be having sex).

**Model 4** : Model 3 plus aspirational variables (assessment of the following in the future: being in a secure job, living with a partner, being in a training scheme, having a child/children, being at college/university, and being in a steady relationship with somebody. Plus an indicator of proportion of friends at other schools and proportion of friends who have left school).

**Model 5** : Model 4 plus factor for school-level deprivation measures.

The following models were also considered in order to assess the influence of individual and school-level socio-economic factors on the outcome variable, independently of pupil attitudes and aspirations:

**Model 6** : Model 1 variables plus factor for school-level deprivation measures.

**Model 7** : Model 2 variables plus factor for school-level deprivation measures.

#### School level data and adjustment for missing data due to attrition at follow-up

Follow-up rates of the original cohorts varied by school. This was largely due to between-school differences in percentages of pupils who had left school at the time of follow up. To investigate the sensitivity of our results to this differential follow-up, two approaches were taken. The first was to omit all school leavers from the analysis, on the basis that schools might be having less impact on these early leavers. However, this analysis excluded a substantial proportion of the original cohort, whose behaviour may have been influenced by the school and would only allow generalization of results to those that remained at school. A better approach was to carry out a weighted analysis to compensate for the pupils missing at follow-up, and thus give inferences that could be applied to the whole original sample.

The weighted analysis assumes that data are missing at random, conditional on the variables used to calculate the weights. Baseline data plus an indicator of early school leaving (overwhelmingly leaving school for good) were used to develop a predictor of whether a pupil would participate at follow-up. The variables included in the weighting were: parental monitoring, family composition, spending money, early school leaving, sex (male/female), social class and level of alcohol consumption. This predictor was then used to calculate an inverse probability weight in order to estimate responses that would have been provided by pupils had they all participated at follow-up. Information for those responders in follow-up was used in the same modelling approach described earlier, with the data weighted using the relevant adjustment for each individual. Thus the school-level predictions arising from each model can be thought of as the proportion of sexually active pupils of each gender, adjusted to the levels that would be expected had the non-responding pupils been surveyed. In the results section we will refer to this as the weighted analysis.

## Results

Overall rates of sexually active pupils for the modelling sample were found to be 42% for girls and 33% for boys. Values by school ranged from 23% (school 14) to 61% (school 12).

### Leavers and the issue of weighted data

The next stage in the analysis was to explore the impact of early school leavers. It was noted that 15% (N = 747) of the sample participating at follow-up had left school, with slightly higher rates for girls than boys (17% and 14% respectively).

Between-school variances arising from models with leavers omitted were found to be similar (generally slightly lower) than those with all pupils included, and the pattern of increases and decreases through the addition of groups of variables remained the same (results not presented). Predicted levels of sexual experience by school decreased somewhat with the removal of school-leavers from the data. Nonetheless, the pattern of 'school effects' remained. To establish to what extent the exclusion of leavers had affected the ranking of schools in terms of predicted proportions of sexually active pupils, rank correlation coefficients were calculated using the rankings of predicted values for schools with leavers included and their rankings once these leavers were removed. In all cases it was found that the rankings of schools based on predictions with and without leavers were highly positively correlated (r = 0.91, p < 0.000 for females and r = 0.94, p < 0.000 for males). This confirmed that the exclusion of leavers did not substantially alter the picture in terms of 'school effects'.

Unweighted analysis generalizes only to those remaining at school. A more appropriate method of taking account of the differential response rate for leavers is a weighted analysis that compensates for the missing pupils. Thus all the results will be presented for weighted analysis, especially since the same pattern of results will be provided.

### Predictors of sexual experience

Odds from fitting weighted Model 4 are provided in Table 2 for variables noted to be significant predictors of sexual experience for either or both genders. Predictors of significance for girls and boys were noted to be: age in months at time of follow-up interview, mother's age, level of personal spending money, level of parental monitoring, adults lived with, attitude to school, expectations for a future steady relationship, the proportion of friends who had already left school, and the proportion of friends perceived to be having sex. In addition, a significant effect was observed for girls in respect of ethnic group and strength of religious belief, and for boys in respect of expectations of living with a partner and of attending college in the future. The results in Table 2 confirm the predictors of sexual experience published previously [28,29].

**Table 2:** Multivariate pupil level predictors of sexual experience among 15 and 16 year old pupils (N = 4925) in 24 Scottish schools (the results that are statistically significant are in bold

**Covariate**	**Sub-group**		**BOYS**			**GIRLS**	
		Odds ratio	(95% CI)		Odds ratio	(95% CI)	
Age in months at follow-up interview		**1.05**	1.02	1.08	**1.07**	1.04	1.09
Adults pupil lives with	Mum only	1.30	0.98	1.72	**1.51**	1.20	1.89
	Dad only	1.47	0.87	2.48	**2.07**	1.16	3.71
	Other	**4.21**	1.17	15.12	1.79	0.59	5.47
	Both parents	1			1		
Parental monitoring	High	**0.73**	0.59	0.91	**0.66**	0.54	0.80
	Low	1			1		
Spending money	High	**1.65**	1.33	2.04	**1.45**	1.20	1.77
	Missing	1.08	0.59	1.97	1.41	0.84	2.36
	Low	1			1		
Ethnic group	Indian subcontinent	0.63	0.25	1.57	**0.26**	0.09	0.78
	Missing	1.04	0.45	2.41	0.65	0.17	2.48
	Other	0.52	0.24	1.14	0.57	0.28	1.14
	White	1			1		
Religious belief	Very religious	0.30	0.07	1.23	0.24	0.05	1.09
	Religious	0.91	0.57	1.44	0.87	0.61	1.26
	not religious	1.14	0.84	1.54	1.19	0.93	1.53
	not at all religious	1.18	0.89	1.57	**1.58**	1.23	2.03
	Unsure	1			1		
Mother's age	Missing	1.05	0.79	1.40	0.85	0.57	1.27
	Under 40	**1.52**	1.19	1.94	**1.40**	1.14	1.73
	40/over 40'	1			1		
Attitude to school (higher score:poorer attitude)		**1.23**	1.08	1.41	**1.41**	1.23	1.62
Future live with partner	Very unlikely	1.00	0.55	1.82	0.84	0.57	1.24
	Unlikely	1.11	0.80	1.53	0.96	0.75	1.22
	Likely	**1.54**	1.16	2.05	1.32	0.99	1.76
	Very likely	**2.13**	1.30	3.50	1.50	0.75	2.99
	Unsure	1			1		
Future college	Very unlikely	**2.73**	1.50	4.99	0.86	0.36	2.06
	Unlikely	1.32	0.85	2.06	**0.57**	0.34	0.94
	Likely	0.94	0.73	1.22	0.81	0.62	1.05
	Very likely	0.83	0.59	1.17	**0.73**	0.53	0.99
	Unsure	1			1		
Future steady relationship	Very unlikely	1.12	0.47	2.67	0.53	0.19	1.46
	Unlikely	1.23	0.67	2.24	0.94	0.60	1.49
	Likely	**1.45**	1.13	1.85	**1.50**	1.22	1.85
	Very likely	**2.74**	1.77	4.24	**2.12**	1.38	3.26
	Unsure	1			1		
Friends left school	Missing	1.70	0.77	3.74	0.91	0.35	2.38
	Most or all	2.31	0.90	5.94	**3.15**	1.57	6.34
	Half	1.86	0.88	3.92	**1.84**	1.02	3.31
	a few	**1.46**	1.17	1.83	**1.77**	1.45	2.18
	None	1			1		
Proportion of friends perceived to be having sex	Missing	**0.62**	0.41	0.93	**0.49**	0.32	0.76
	None	**0.33**	0.13	0.84	**0.02**	0.00	0.25
	Very few	**0.50**	0.35	0.71	**0.61**	0.42	0.88
	About a quarter	0.84	0.58	1.21	**0.67**	0.48	0.93
	Less than half	0.88	0.64	1.21	0.85	0.64	1.13
	Most of them	**1.44**	1.06	1.94	**1.34**	1.05	1.72
	all of them	**1.12**	0.40	3.11	**4.82**	1.46	15.95
	Half	1			1		

### Comparison of Models

School variance parameters (school effects) were produced as part of the output for each of the models investigated (Table 3). Sizes of between-school variances relative to Model 1 were of interest here, and percentages in brackets are the proportion of each figure relative to its relevant Model 1 result.

**Table 3:** Residual between-school variance in sexual experience among 15 and 16 year old pupils (N = 4925) in 24 Scottish schools, controlling for individual level and area-level variables (weighted analysis)

	Girls		Boys	
**Model 1 **– pupils' age in months at follow-up and cohort	0.129	(100%)	0.174	(100%)
**Model 2 **– This model adds (to Model 1) pupils' individual socio-cultural variables (e.g. family composition and parental monitoring)	0.027	(21%)	0.080	(46%)
**Model 3 **– This model adds (to Model 2) attitudinal variables (e.g. attitude to school)	0.039	(30%)	0.096	(55%)
**Model 4 **– This model adds (to Model 3) aspirational variables (e.g. future univerity/college course)	0.048	(37%)	0.116	(67%)
**Model 5 **– This model adds (to Model 4) the factor for school-level deprivation measures	0.036	(28%)	0.075	(43%)
**Model 6 **– This model adds (to Model 1) the factor for school level deprivation measures	0.021	(16%)	0.049	(28%)
**Model 7 **– This model adds (to Model 2) the school level deprivation measures	0.016	(12%)	0.042	(23%)

From this Table 3 it can be seen that in Model 2 the addition of individual-level socio-economic variables to the base model produced a sizeable reduction in the school variance component. Looking at the addition of the group of attitude variables in Model 3, it can be seen that for both genders between-school variance increased slightly. A further increase was also observed in Model 4 following the addition of aspiration variables. These results were surprising, as we had expected that schools might have influenced attitudes and aspirations and that these would thus be part of the causal mechanism that produced the between-school differences. However, it appears that this is not happening and that, while schools may have influenced attitudes, there has not been a corresponding change in behaviour usually associated with these attitudes.

It was noted that the inclusion of a school-level composite variable reflecting deprivation in Model 5 reduced the between-school variation for both girls and boys, but particularly so for boys. The exclusion of pupils' attitudes and aspirations whilst including school-level deprivation information (Models 6 and 7), reduced the variance below that observed in any of the other models. This indicates that the differences in levels of sexual activity between schools could largely be attributed to individual and school-level socio-economic factors.

#### Predictions

**Figure 1:**
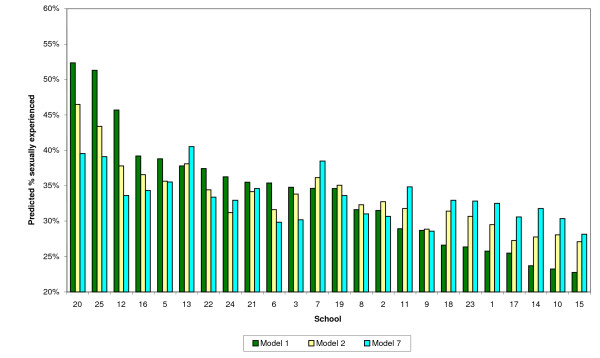
Comparison of models 1, 2 & 7 – boys (weighted).

School-level predictions were generated using values for schools arising from each model to adjust the proportion of sexually active pupils for each gender. Results presented in Figures 1 (males) and 2 (females) and Table 3.

**Figure 2:**
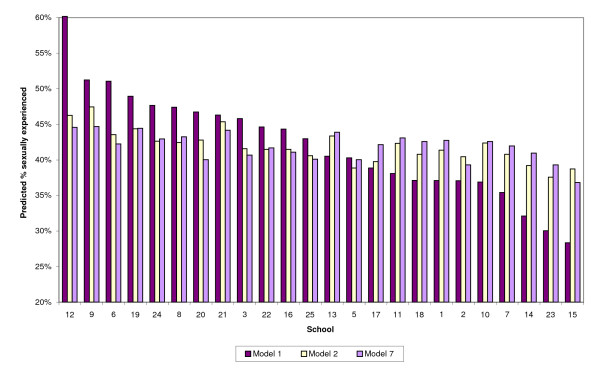
Comparison of models 1, 2 & 7 – girls (weighted).

The Figures (1 &2 below) show predicted percentages of sexually active pupils for schools from Models 1, 2 and 7. These are displayed on a scale from 20% to 60% for clarity and ease of comparison. Each figure is in order of predicted levels of sexual experience for Model 1, again for ease of interpretation.

The figures illustrate how differences in predicted values for the models are generally reduced with the inclusion of individual socio-cultural factors (Model 2), and flatten out with the inclusion of school-level characteristics (Model 7).

**Figure 3:**
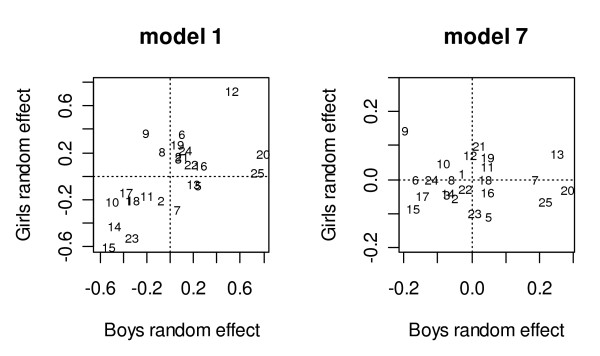
Random effects labelled by school number and using weighted residuals.

School residuals for boys and girls from Model 7 were plotted against one another in order to highlight schools with unusual results. This is shown in Figure 3, alongside a plot of residuals from Model 1.

Comparing the two plots in Figure 3 shows that socio-economic factors (included Model 7's plot) seem to account for girls' between-school variation much more than for boys'. Also, when individual- and school-level socio-economic factors have been taken into account (Model 7 plot), in general schools are clustered together in the centre of the picture, indicating that for the majority there is little to be seen in the way of unusual 'school effects'.

Schools appearing as outliers (Schools 13, 7, 25, 20, 15 and 9) were highlighted in order to explore whether their positions could be explained by the process data. These schools were located furthest from the origin in Model 7's plot (see Figure 3), indicating that the school effect was either higher or lower for girls or boys (or both) than might be expected after known predictors had been accounted for. Four out of six of these outlying schools were located in large or small towns as opposed to cities, compared with 11 out of 24 for the whole sample.

### Process data

The process data were being used to explore whether they can help explain the 'school effects' (e.g. why are the outlying schools different to the other schools?). The process data were presented in order of Model 7 outcomes for males (the ranks for females are also shown), schools with the best outcomes for males coming first (Table 1). The scores that are above average are boldened in Table 1.

Schools 13, 7, 25, 20, 15 and 9 were noted from Figure 3 (model 7's plot) to have larger residuals/variance. There is no obvious pattern between these school residuals and the process data. For instance, the Health Promoting School model would predict poor processes for School 20 as it has the highest rates of sexual experience for boys and just below average rate for girls. The processes were indeed poor in that it scored poorer than average in all of the process dimensions, apart from the appearance of the school. School 9 (in the same town) which scored average on all the dimensions apart from the layout of the school and physical environment, partially fits the model for boys (as boys had lowest school rates of sexual experience), but was completely counter it for girls (as girls had highest school rates of sexual experience). The other outlying schools have very mixed process data. Approaching this analysis conversely, the school with the worst process scores, which were independently corroborated by a damning report from Her Majesty's Inspectorate of Schools (School 5), had the lowest rate of sexual experience for girls and an average rate for boys.

A further way of exploring the data was to compare the 12 schools with lowest residual levels of sexual experience with the 12 schools with highest residual levels of sexual experience for males and females separately. Again, this revealed no clear patterns other than the following: of the 12 schools with lowest levels of sexual experience for boys, 10 had high ratings of classroom discipline, as measured through researchers' perceptions, compared with 4 of the 12 schools with high levels of sexual experience. For girls, the only finding of note is that only 2 of 12 schools with low levels of sexual experience had above average scores for pupil-pupil relationships, compared with 9 of 12 schools with high levels of sexual experience.

## Discussion

There was considerable variation between schools in rates of sexual experience at average age 16. This was expected given the association between sexual experience and smoking [35] and the results of similar analyses on smoking [21-23]. Involvement in school by staying on beyond the minimum leaving age was associated with lower rates of sexual experience and this is in line with previous research [25]. However, when individual socio-economic and cultural factors were taken into account this variation dropped sharply. School-level socio-economic factors had an additional but smaller effect in reducing the variation between schools. The fact that school-level socio-economic factors are predictive even after taking account of individual socio-cultural factors suggests that the wider socio-economic environment that young people inhabit further influences their sexual experience. Therefore, being individually deprived but attending a school with an affluent catchment may discourage sexual activity, whilst being affluent and attending a school with deprived catchment may encourage earlier sexual intercourse.

The process data presented in this paper did not adequately explain why some schools were doing particularly well and others particularly badly with regard to the residual variation between schools in sexual experience. For this particular outcome we can present only very weak evidence for the HPS model, and this only holds for males. Stronger evidence had been expected given the results for other evaluations of HPS in relation to smoking, [21,22] drug use, [23,24] mental health and aggression [18]. Beyond deprivation, characteristics of the school may have less influence on sexual experience than other factors such as peer groups, neighbourhood culture or the youth-friendliness and accessibility of local family planning provision [35].

Despite having robust measures of teacher-pupil relationships, the quality of these relationships was not associated with levels of sexual activity. Similarly, the lack of influence of attitudes and aspirations to explain 'school effects' was notable and these findings raise further issues. Perhaps the lack of explanatory value of quality of relationships, attitudes and aspirations indicates that young people's sexual behaviour is influenced far more by background socio-cultural factors that they have little influence over, although there is a tension between the evidence at this group level and that at individual level (Table 2). If so, this may have important policy implications: for instance, that young women should be empowered to pursue their aspirations even when background socio-cultural factors have led them to have sex early.

The within-school process data were not collected purposely to explain 'school effects' or test the HPS concept, and it may be that their inability to do so reflects their inadequacy, particularly in being largely restricted to Personal and Social Education lessons and staff, rather than representing pupils' whole school experience. The process data collected did not allow us to explore school level consistency and cohesion across classes and departments, constancy over time, or control, factors considered to be important to a wide range of student outcomes, [12] so they may have helped explain our findings.

The results of this study are clearly gendered, with very little correlation between boys' and girls' rates of sexual experience by school, once individual- and school-level socio-economic variables have been taken into account. This contrasts with results from the base model (Model 1). In the weighted analysis the lowest between-school variance was in Model 7 (where individual socio-cultural and school-level deprivation were included), for both boys and girls. In the unweighted analysis the same result was observed for boys but not for girls (unweighted results not presented). This suggests that re-weighting the girls' data increases the power of the individual socio-cultural factors, and confirms the idea that 'school effects' on sexual experience are strongly influenced by both individual socio-cultural factors as well as school-level deprivation for both boys and girls. The data further suggest that girls are more influenced by individual socio-cultural and socio-economic factors than boys. The only patterns that could be discerned linking process data to outcomes were both gender-specific.

The quantitative data show that two schools in the same town have very different levels of predicted sexual activity by gender (Schools 9 & 20), and these schools have very different process data. School 20 was scored average or below average on all process dimensions, apart from appearance. School 9 was scored average or above on all process dimensions, apart from appearance. Since the schools are in the same conurbation with similar catchment areas, this suggests that the contrasting levels of sexual activity might be related to processes within the schools. School 9 is interesting as it has the best outcome for boys and poorest outcome for girls. This suggests that the within-school processes that might affect sexual experience are different for boys and girls. Greater classroom discipline, as observed by researchers, is associated with lower levels of sexual experience for boys, but the mechanism for this is not clear. For instance the results could be interpreted as meaning either classroom discipline actually discourages early sexual activity or that classroom discipline reduces the reporting of sexual experience. For girls, better pupil-pupil relationships, as observed by researchers, are associated with higher levels of sexual experience. The latter finding might be due to greater peer pressure among girls when pupil-pupil relationships are stronger. These findings illustrate the importance of gender, although the process measures have some limitations.

There is evidence for effects of neighbourhood culture. Large city schools have the smallest residuals for both boys and girls, but particularly for boys (see Figure 3, model 7's plot where these schools can be seen to cluster around the centre of the plot). For girls, Dundee schools (in Tayside) tend to have slightly higher levels of sexual activity than in the Edinburgh schools (in Lothian); this is confirmed externally by the higher rates of teenage pregnancy in Dundee (Information and Statistics Division, 2000). Outlying schools tend to be in towns rather than cities (Figure 3, model 7's plot).

Future research might address the limitations of our process measures by collecting more systematic data on school-wide processes that might influence sexual behaviour. It would also be beneficial to explore whether schools have different effects on different sub-groups of pupils, and to explore in more depth the impact of classroom discipline on young men and pupil-pupil relationships on young women.

## Conclusion

To our knowledge, this is the most rigorous attempt to establish whether schools influence pupils' rates of sexual experience other than through the formal curriculum, and if so, by what processes. The paper establishes that there is variation between schools in levels of early sexual experience, and that much of this, particularly for girls, is explained by both individual and school level socio-economic factors. The remaining variance is not explained by a robust measure of pupil-teacher relationships, nor by several other measures of school processes, although there is a suggestion that classroom discipline might influence boys and pupil-pupil relationships girls. However, from these findings it seems that between-school variance in rates of reported sexual experience, after controlling for known predictors, is likely to owe more to neighbourhood culture than within-school processes. Further research is needed to establish an appropriate policy response to these findings.

## Competing interests

The author(s) declare that they have no competing interests.

## Authors' contributions

MH, DW and GR designed the original study, while MH, DW, IB & GR collected the data. IB, MH, LW and GR analysed the data. DW commented on the analysis. MH and IB drafted the paper and MH revised subsequent drafts based on co-authors' comments. MH, IB, LW, DW and GR commented on subsequent drafts of the paper. All authors read and approved the final manuscript.

## Pre-publication history

The pre-publication history for this paper can be accessed here:



## References

[B1] Currie C, Todd J (1993). Health Behaviours of Scottish Schoolchildren: Report 3: Sex Education, Personal Relationships, Sexual Behaviour and HIV/AIDS Knowledge and Attitudes.

[B2] Graham A, Green L, Glasier A (1996). Teenagers' knowledge of emergency contraception: questionnaire survey in South East Scotland. British Medical Journal.

[B3] HMSO (1992). The health of the nation: a strategy for health in England (White Paper, CM 1986).

[B4] Scottish Office Department of Health (1999). Towards a healthier Scotland: a White Paper on health.

[B5] Social Exclusion Unit (1999). Teenage Pregnancy.

[B6] Wight D, Raab G, Henderson M, Abraham C, Buston K, Hart G, Scott S (2002). The limits of teacher-delivered sex education: interim behavioural outcomes from a randomised trial. British Medical Journal.

[B7] Fitz-Gibbon CT, Hopkins D and Reynolds D (1996). Monitoring education: indicators, quality and effectiveness. School Development Series.

[B8] Coleman JS, Campbell EQ, Hobson CF, McPartland J, Mood AM, Weinfeld FD, York RL (1966). Equality of Educational Opportunity.

[B9] Reynolds D, Jones D, St Leger S (1976). Schools do make a difference. New Society.

[B10] Rutter M, Maughan B, Mortimore P, Ouston J (1979). Fifteen Thousand Hours.

[B11] Reynolds D, Sammons P, Stoll L, Barber M, Hillman J (1996). School Effectiveness and School Improvement in the United-Kingdom. School Effectiveness and School Improvement.

[B12] Reynolds D, Bollen R, Creemers B, Hopkins D, Stoll L, Lagerweij N (1996). Making good schools: linking school effectiveness and school improvement.

[B13] World Health Organisation, Council of Europe, Commission of European Communities (1993). The European Network of Health Promoting Schools: a joint World Health Organisation - Council of Europe - Commission of European Communities project.

[B14] Gordon J, Turner K (2001). School staff as exemplars - where is the potential?. Health Education.

[B15] Parsons C, Stears D, Thomas C, Thomas L, Holland J (1997). The implementation of the European Network of Health Promoting Schools (ENHPS) in different national contexts.

[B16] Mortimore P, Sammons P, Stoll L, Lewis D, Ecob R (1988). School matters: the junior years.

[B17] MacBeath J, Thomson B, Arrowsmith J, Forbes D (1992). Using ethos indicators in secondary school self-evaluation: taking account of pupils, parents and teachers.

[B18] Health Evidence Network (HEN) (2006). What is the evidence on school health promotion in improving health or preventing disease and, specifically, what is the effectiveness of the health promoting schools approach?.

[B19] Scottish Health Promoting Schools Unit (2004). Being Well - Doing Well: a framework for health promoting schools in Scotland.

[B20] Department for Education and Employment (1999). National Healthy School Standard: Guidance.

[B21] Aveyard P, Markham WA, Cheng KK (2004). A methodological and substantive review of the evidence that schools cause pupils to smoke. Social Science and Medicine.

[B22] Aveyard P, Markham WA, Lancashire E, Almond J, Griffiths R, Cheng KK (2005). Is inter-school variation in smoking uptake and cessation due to differences in pupil composition? A cohort study. Health and Place.

[B23] West P, Sweeting H, Leyland A (2004). School effects of pupils' health behaviours: evidence in support of the health promoting school. Research Papers in Education.

[B24] Henderson M (2006). School effects on adolescent pupils' health behaviours and school processes associated with these effects. MRC Social & Public Health Sciences Unit.

[B25] Kirby D (2002). The impact of schools and school programs upon adolescent sexual behaviour. The Journal of Sex Research.

[B26] Resnick MD, Bearman PS, Blum RW, Bauman KE, Harris KM, Jones J, Tabor J, Beuhring T, Sieving RE, Shew M, Ireland M, Bearinger LH, Udry JR (1997). Protecting adolescents from harm: findings from the National Longitudinal Study on Adolescent Health. Journal of Americn Medical Association.

[B27] Basen-Engquist K, Coyle KK, Parcel GS, Kirby D, Banspach SW, Carvajal SC, Baumler E (2001). Schoolwide effects of a multicomponent HIV, STD, and pregnancy prevention program for high school students. Health Education & Behaviour.

[B28] Henderson M, Wight D, Raab G, Abraham C, Buston K, Hart G, Scott S (2002). Heterosexual risk behaviour among young teenagers in Scotland. Journal of Adolescence.

[B29] Wight D, Henderson M, Burtney E and Duffy M (2004). The diversity of young people's heterosexual behaviour. Young people and sexual health: individual, social and policy.

[B30] Wight D, Dixon H (2004). SHARE: the rationale, principles and content of a research-based teacher-led sex education programme. Education and Health.

[B31] SHARE study public domain Internet site. http://www.sphsu.mrc.ac.uk/studies/share.

[B32] Wight D, Obasi A, Stephenson J, Imrie J and Bonell C (2003). Unpacking the 'black box': the importance of process data to explain outcomes. Effective sexual health interventions: issues in experimental evaluation.

[B33] 
Raab 
G, 
Butcher 
I (2001). Balance in cluster randomized trials. Statistics in Medicine.

[B34] SAS Institute Inc. (1999). SAS Version 8.1, statistical software.

[B35] Poulin C, Graham L (2001). The association between substance use, unplanned sexual intercourse and other sexual behaviours among adolescent students. Addiction.

[B36] Parkes A, Wight D, Henderson M (2004). Teenagers' use of sexual health services: perceived need, knowledge and ability to access. Journal of Family Planning and Reproductive Health Care.

